# Research trends and hotspots in the immune microenvironment related to osteosarcoma and tumor cell aging: a bibliometric and visualization study

**DOI:** 10.3389/fendo.2023.1289319

**Published:** 2023-11-08

**Authors:** Wenlong Zhang, Zhuce Shao

**Affiliations:** ^1^ Shanxi Bethune Hospital, Shanxi Academy of Medical Sciences, Tongji Shanxi Hospital, Third Hospital of Shanxi Medical University, Taiyuan, China; ^2^ Tongji Hospital, Tongji Medical College, Huazhong University of Science and Technology, Wuhan, China

**Keywords:** osteosarcoma, microenvironment, bibliometrics, visualization, MSC, RNA

## Abstract

**Background:**

It is well known that cancers have a common feature that even if the environment is extremely poor in nutrients, they can still make good use of them to maintain viability as well as to produce new biomass, which is one of the reasons why tumor cells are powerfully less susceptible to senescence and death. The microenvironment has a profound impact on the senescence as well as the growth and development of tumor cells, and it is also the focus of scientists’ research because it may even affect the discovery of the treatment and pathogenesis of cancer. And so the study of the microenvironment in the tumor cells is of great significance to the analysis of the tumor cells as well as to the impact of their senescence. Similarly, the microenvironment of osteosarcoma is also crucial for its impact, but to our knowledge, there is no bibliometric study that systematically analyzes and describes the trends and future hotspots in this field of research as we do, and we are going to fill this gap in this study.

**Methods:**

We searched the Web Science Core Collection 2010-2023 in WOS on August 1, 2023. Based on the criteria needed for the search, we retained articles that matched the topic, excluded studies other than articles and reviews, and selected only studies whose language was English. We performed an intuitive visualization and bibliometric approach to analyze the research content in this field and a systematic visualization of global trends and hotspots in the research of osteosarcoma and the microenvironment, for which we used multiple specialized For this purpose, we used several specialized software packages, such as VOSviewer and the Bibliometrix package for R software. Because research in this area of osteosarcoma and the microenvironment has begun to gain popularity in the last 10 years or so, and is a very novel piece of research, there were almost no studies in this area prior to 2010 and they were not very informative, and in the end, we chose to look at studies from after 2010.

**Results:**

Based on the criteria needed for the search, resulting in a final selection of 821 articles. In the research area related to osteosarcoma and microenvironment, we found that China in Asia and the United States in North America and Italy in Europe were the three countries or regions with the highest number of published articles. In addition, the institution that published the most research in this area was Shanghai Jiao Tong University. In terms of publications in the field of osteosarcoma and microenvironmental research, Baldini, Heymann, and Avnet are among the top 3 authors. The terms “cancer”, “cells” and “expression” are found to be more commonly employed.

**Conclusion:**

Using a variety of highly specialized software, we have undertaken a visual and bibliometric study of the current state of research and potential future hotspots in the field of osteosarcoma and microenvironment research. The microenvironment has a profound impact on the senescence and growth and development of cells in tumors, including osteosarcoma, and may even influence the discovery of cancer treatment and pathogenesis, and is also a hotspot and focus that scientists have begun to gradually study in recent years. This analysis and visualization will help guide future research in the field.

## Introduction

The internal environment in which tumor cells grow and survive is referred to as the tumor microenvironment, or TME. The extracellular matrix’s non-cellular components, such as cytokines and chemokines, as well as the surrounding fibroblasts, immune and inflammatory cells, vascular cells, and other stromal and immune cells linked to cancer are all included in the microenvironment in addition to the tumor cells themselves (1, 2), The relationship between the tumor and its microenvironment has drawn increasing attention from academics over the course of so many years of research. Especially at the present stage when the tumor treatment has come to a bottleneck, and the recurrence and prognosis of cancer have become a headache. At this time, many people believe that the microenvironment may be one of the keys to solving these thorny problems, and many studies have proved that there is a great connection between the development and recurrence of cancer and its microenvironment (3–5).The most important features of the tumor microenvironment are hypoxia, chronic inflammation, and immunosuppression. They work in harmony with one another to support the growth and development of tumor cells as a whole.

A very aggressive bone tumor with an annual incidence of about 480 per million people, osteosarcoma is more common in children and adolescents (6, 7). Despite an increase in treatment choices, patients with osteosarcoma still have a 5-year overall survival rate that is less than 70% (8). And the recurrence rate remains around 30%. Researchers have found that the poor prognosis and recurrence of osteosarcoma may be the paucity of knowledge of the molecular pathways underlying the onset and spread of osteosarcoma, researchers discovered that the mesenchymal stem cells (MSCs) in the osteosarcoma microenvironment, in particular, may be directly associated to osteosarcoma development and metastasis. In a study of osteosarcoma in rats, it was found that MSC significantly promoted lung metastasis of osteosarcoma (9). According to the findings of numerous research, the rejection of TP53 and Rb causes osteoblasts to develop into osteosarcoma cells (10–12). Loss of Rb may lead to further malignant transformation of MSC into osteosarcoma, and overexpression of C-myc may cause MSC to have a similar outcome to Rb loss (13).

The tumor microenvironment is the most favorable site for osteosarcoma growth and development and metastasis. If the microenvironment of bone can be more deeply understood, the mechanisms of osteosarcoma development as well as metastasis can be better comprehended, and furthermore, targets to stop or reduce OS metastasis can be found to solve the problem at the root.

To undertake a rigorous quantitative and qualitative study of the studies on osteosarcoma and the microenvironment, we will employ bibliometrics. To better direct the research in this sector, bibliometrics can more clearly display the hotspots of that research as well as the direction of potential future cutting-edge trends (14, 15).

## Materials and methods

### Process of gathering and retrieving data

**Figure 1:**
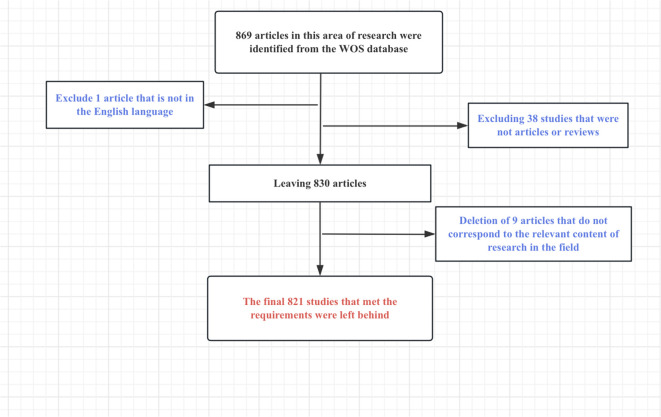
Flowchart for screening articles that meet the requirements.

We systematically searched the Web of Science (WoS) from January 1, 2020, to August 1, 2023. It is well known that WoS contains almost the richest category of journals and is the most read and frequently read database for specialized sciences (16). The WoS database is more scientific, specialized and comprehensive than some other databases (17). There is no doubt that the WoS database is best suited for bibliometric research analysis (18–20). We also downloaded the data on 8/1/2023. Search terms included TS= (osteosarcoma) and TS= (microenvironment). The data were saved in txt format after downloading, which were plain text files, preserving the complete record and citing references. After we scrutinized and screened several times, only papers and review articles were retained, literature not related to the research in this field was excluded. Also, it should be emphasized that we are choosing “Science Citation Index Expanded” and “Social Sciences Citation Index” for WoS. The entire procedure of screening the literature is effectively shown in **Figure 1**.

## Results

### Information about published studies that are generally related to this topic of osteosarcoma and the microenvironment

**Figure 2:**
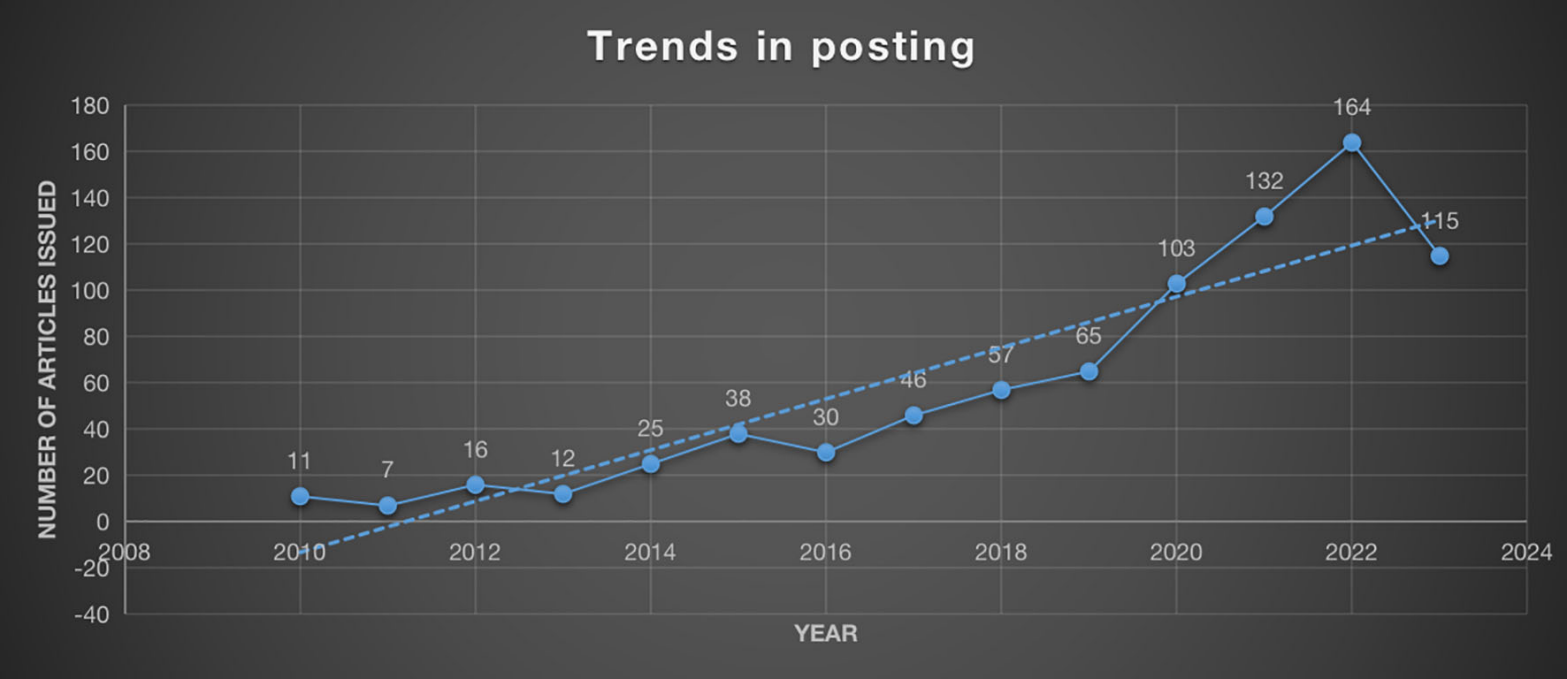
Trends in the volume of publications published each year in the study of the microenvironment and osteosarcoma.

From the findings of all the research on osteosarcoma and the microenvironment that has been published, the final results show a total of 821 research articles, of which they are included in 304 journals, 4839 authors have conducted research in the field of Osteosarcoma and Microenvironment, and for the 13-year period from 2010 to 2023, we found that a total of 1105 institutions and 55 countries are involved in researching in the field. Publications in the field of osteosarcoma metabolism cited 40,533 articles from 3,817 journals. Of these, out of a total of 821 studies in the field, 417 were found to be from China, 170 from the United States, and 76 articles from Italy, which were the top three countries or regions with the most research publications in the field. France, Japan, and England are the top 4-6 nations with the most publications in this area. Despite having only 39 papers published, England came in top place with an average of 31.97 citations, suggesting that the country’s research in this area is more well-known. **Table 1** lists the top 10 countries with the highest number of published articles in osteosarcoma and microenvironment research. In addition, as can be seen in **Figure 2**, the publication of papers in osteosarcoma and microenvironment-related research demonstrates a general trend of steady increase. Of course, since we are still counting that data on August 01, 2023, the number of articles published at that point is 115, but the prediction for 2023 based on the first two quarters of publications is that 170 articles in this area of research will be published in 2023, so the overall trend is still upward.

**Table 1:** Top 10 nations in terms of publications in the topic of microenvironment and osteosarcoma.

ID	Country	Documents	Citations	Average number of citations
1	Peoples r china	416	6681	16.06
2	Usa	170	4668	27.46
3	Italy	76	2139	28.14
4	France	49	1462	29.84
5	Japan	44	1358	30.86
6	England	39	1247	31.97
7	Australia	29	511	17.62
8	Germany	22	358	16.27
9	Spain	19	549	28.89
10	India	14	257	18.36

### Visual analysis of country or region collaboration in the study of osteosarcoma and the microenvironment worldwide, 2010 to 2023

**Figure 3:**
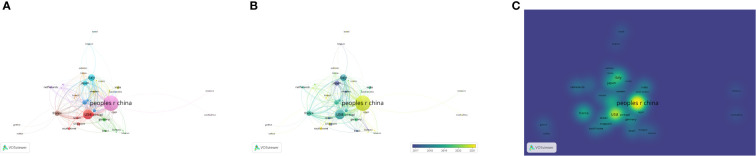
Different representations of the graphs of cooperation relationships between countries or regions, namely the network of such relationships, the graph of such relationships with change over time, and the graph of such relationships’ density, are shown in **(A-C)**, respectively.

**Figure 4:**
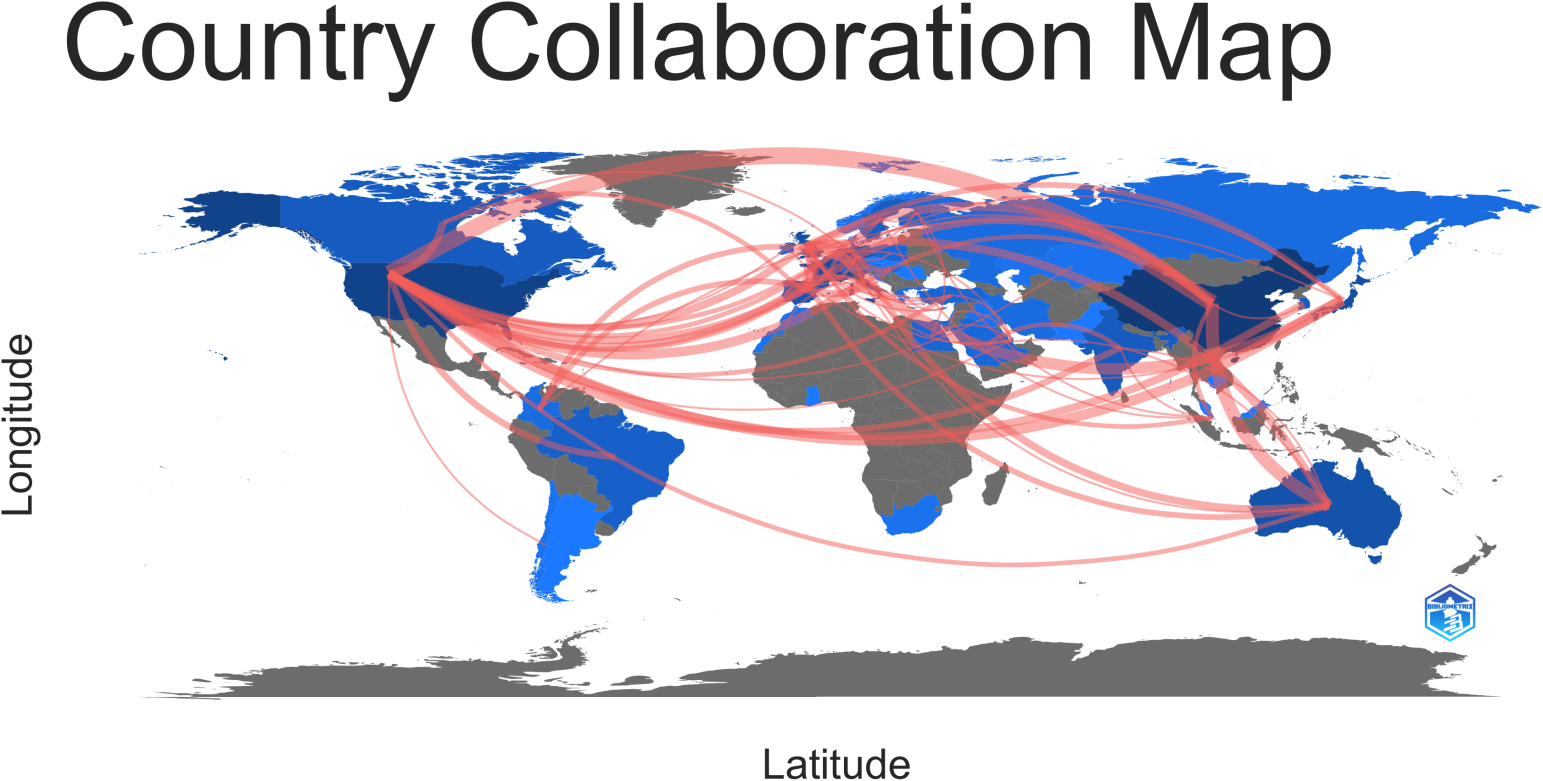
Using the Bibliometrix tool for the R programming language, national and regional cooperation in the fields of osteosarcoma and microenvironmental research were geographically shown.

**Figure 3** provides a visual representation of the network of international collaborations in the study of osteosarcoma and the microenvironment from January 1, 2010, to August 1, 2023. As shown in **Figure 3** and according to **Table 1**, a total of 55 countries or regions from around the world have participated in studies on osteosarcoma and the microenvironment over the course of the past 13 years. These countries or regions have also collaborated on this research area quite frequently. More than 20 papers in this field of study have been published in a total of 8 nations or regions. It is clear from **Figure 3**’s depiction of the cooperation patterns among the various nations and regions involved in osteosarcoma and microenvironment research that the United States and China are the field’s absolute hubs, with other nations picking up on these trends as they radiate outward from their respective centers in China and elsewhere. Different representations of the graphs of cooperation relationships between countries or regions, namely the network of such relationships, the graph of such relationships with change over time, and the graph of such relationships’ density, are shown in **Figures 3A-C**, respectively. As shown in **Figure 4**, to better show the collaboration between countries or regions, we again used the Bibliometrix package in R to create a collaboration diagram similar to a world map for the research field of osteosarcoma and microenvironment.

### Collaborations among authors who published articles in research areas related to osteosarcoma and the microenvironment from 2010 to 2023 were visualized and analyzed using VOSviewer software

We used VOSviewer software to analyze the articles published between 2010 and 2023 in this field of study on osteosarcoma and the microenvironment. We discovered that 4839 authors in total are involved in this field of study. We also visualized the author collaborations with the top 92 authors who have published the most articles in this field. Each of these 92 authors has published at least four or more articles, as calculated by Price Law, m=0.749*√n_max_=3.432 (n_max_=21), and the detailed information of the authors who are ranked in the top 12 in terms of the number of articles published in the research in the field of Osteosarcoma and Microenvironment is shown in **Table 2**.

**Table 2:** top 12 authors with the highest number of publications in research areas related to osteosarcoma and the microenvironment and their article citations.

ID	Authors	Documents	Citations	Average number of citations
1	Baldini, nicola	21	854	40.67
2	Heymann, dominique	19	537	28.26
3	Avnet, sofia	18	765	42.50
4	Redini, francoise	18	650	36.11
5	Guo, wei	11	366	33.27
6	Ren, tingting	10	345	34.50
7	Verrecchia, franck	10	505	50.50
8	Cortini, margherita	9	332	36.89
9	Huang, yi	9	393	43.67
10	Kleinerman, eugenie s.	9	326	36.22
11	Perut, francesca	9	316	35.11
12	Wang, wei	9	210	23.33

**Figure 5:**
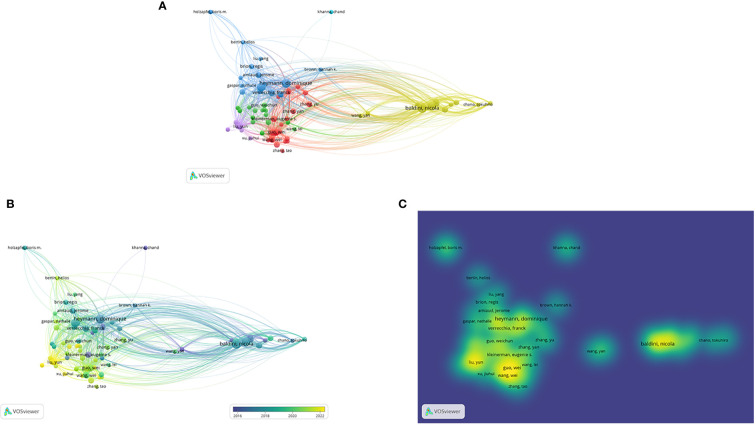
**(A-C)** are the graphs of collaborations between authors, changes in author collaborations over time, and changes in the heat and density of author collaborations, respectively.

From **Figure 5**, we can find that global collaborations are becoming increasingly close in the field of research on osteosarcoma and microenvironment, **Figures 5A-C** are the graphs of collaborations between authors, changes in author collaborations over time, and changes in the heat and density of author collaborations, respectively.

The four authors, Baldini, Heymann, Avnet, and Redini, who conducted the study of osteosarcoma and microenvironment, have published a large number of studies related to this field, and are the more cutting-edge and hot researchers in this field. Among them, we learned after analysis that Baldini’s multiple acts involved the influence of the acidic microenvironment in osteosarcoma on the development of osteosarcoma (16, 21). The role of mesenchymal stroma on osteosarcoma as well as its microenvironment was also investigated (17, 18). In total, he produced 21 publications in the topic, with an average of 40.67 citations per. These 21 papers received 854 citations overall. Verrecchia, Franck also has the highest average number of citations in the field, with 50.50, despite not being the most prolific author of published research in the area. His articles have frequently described the past, present, and future of the intricate ecosystem known as the osteosarcoma microenvironment (19, 20).

### Analysis of various institutions publishing research on osteosarcoma and microenvironment

With a total of 42 articles on this study topic and a total of 1,521 citations, VOSviewer results demonstrate that Shanghai Jiao Tong University ranks first among all academic institutions globally in terms of the number of publications in the subject of osteosarcoma and microenvironment. Additionally, in terms of the quantity of articles produced by their organizations in this sector, Central South University and University of Bologna were rated second and third globally, respectively. The top ten institutions in the world according to the quantity of this field’s publications produced are detailed in **Table 3**.

**Table 3:** Top 10 institutions with the highest number of publications in research areas related to osteosarcoma and the microenvironment and their article citations.

ID	Organization	Documents	Citations	Average number of citations
1	Shanghai jiao tong univ	42	1521	36.21
2	Univ bologna	26	757	29.12
3	Cent south univ	25	172	6.88
4	Huazhong univ sci & technol	25	353	14.12
5	Univ nantes	25	873	34.92
6	Sun yat sen univ	23	341	14.83
7	Zhejiang univ	22	372	16.91
8	China med univ	19	444	23.37
9	Chinese acad sci	19	832	43.79
10	Zhengzhou univ	19	126	6.63

**Figure 6:**
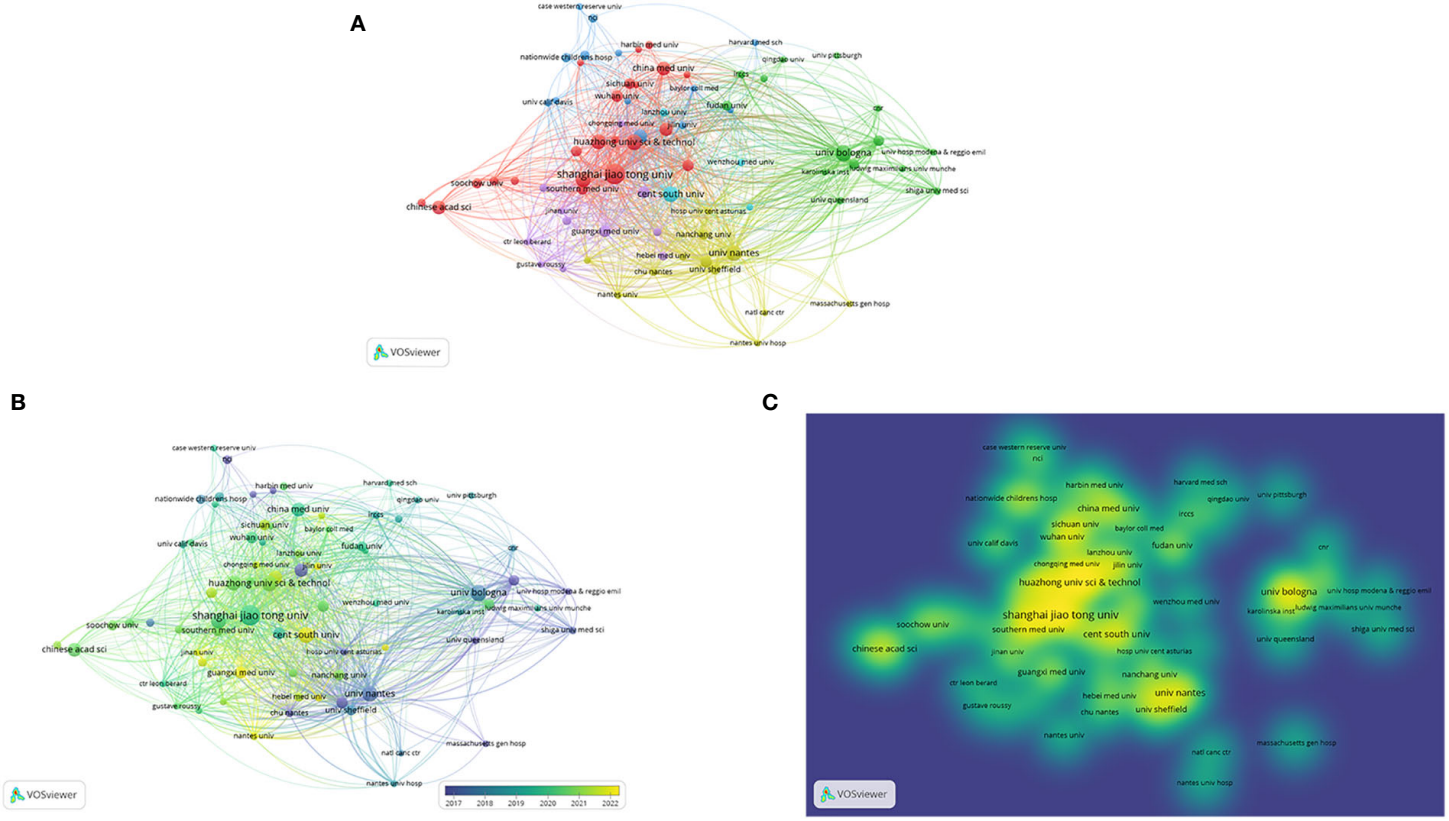
**(A-C)** show the linkages between institutions, the variation of institutions’ collaborations over time, and the variation of institutions’ collaboration density and hotspots, respectively.

**Figure 6** is a visualization of the collaborations of the major global institutions that publish articles in this research area, and we can find that Shanghai Jiao Tong University, Central South University, and University of Bologna are at the center and have close collaborations with many other institutions. **Figures 6A-C** show the linkages between institutions, the variation of institutions’ collaborations over time, and the variation of institutions’ collaboration density and hotspots, respectively.

### Numerous studies on osteosarcoma and microenvironment research have been published in various journals

**Figure 7:**
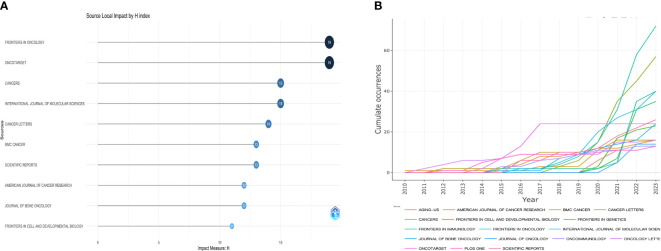
Trend of the top 10 journals in terms of H-index and the top 15 journals in terms of the quantity of articles published. **(A, B)** displays, respectively, the H-index ranking of journals in the discipline and the trend in the number of papers published in those journals over time.

The top ten journals in terms of H-index among all journals ranked in this area of research for the years 2010 to 2023 are presented in **Figure 7A** as a result of VOSViewer software’s analysis of the research area of Osteosarcoma and Microenvironment. The trend in the volume of articles published in the top 15 journals in the field over the past few years is very clearly displayed in **Figure 7B**.

### In-depth analysis of keywords in the field of osteosarcoma and microenvironment research

**Figure 8:**
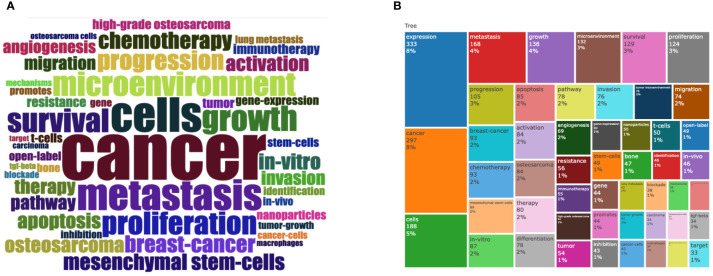
The presentation of keywords for research in this field. **(A, B)** shows the different representations of the percentage of keywords researched in the field.

It is well recognized that a study field’s rapidly expanding term usage can serve as a reliable predictor of the field’s current hot trend and future hot direction. The keywords with the highest frequency in the research field of osteosarcoma metabolism from 2010 to 2023 are shown in **Figures 8A, B**. Their sizes can be used to denote their importance and high frequency of use in the field. We analyzed the keywords in the research field of osteosarcoma and microenvironment using the Bibliometrix package in R.

**Figure 9:**
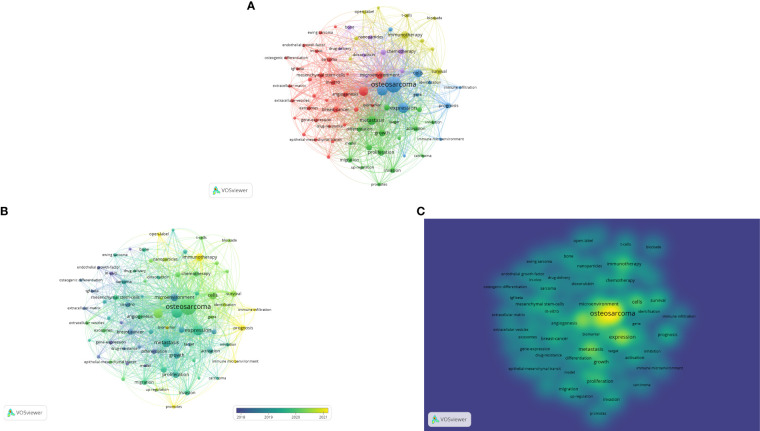
**(A-C)** illustrate the linkages between keywords, the evolution of keyword linkages over time, and the changes in keyword density and linkages over time, respectively.

The phrases “cancer,” “cells,” and “expression” are found to be used more frequently, which reflects the field’s current and potential future directions. This shows the direction in which the discipline is currently and potentially going to evolve. **Figures 9A–C** illustrate the relationship between keywords, the evolution of keywords through time, and the variation in keyword density, respectively.

### Analysis of keywords exploding in osteosarcoma and microenvironment research 2010-2023

One of CiteSpace’s distinctive features is its ability to highlight potential hotspots and trends that may have formed over each time period by displaying the abrupt proliferation of terms in a given field of study during a certain time period. As seen in **Figure 8**, we employed the software to assess the verbal boom in the area of osteosarcoma metabolism. A sudden explosion in that time period is shown by the red hue on the line that follows each outburst period. It is generally accepted that an outbreak of words in a particular field of study is indicated to be of some significance when the intensity of the words is greater than, say, 3.0, and we found that many of the words in this study of the osteosarcoma microenvironment met this requirement, which is indicative of the hotness of the research in this field.

**Figure 10:**
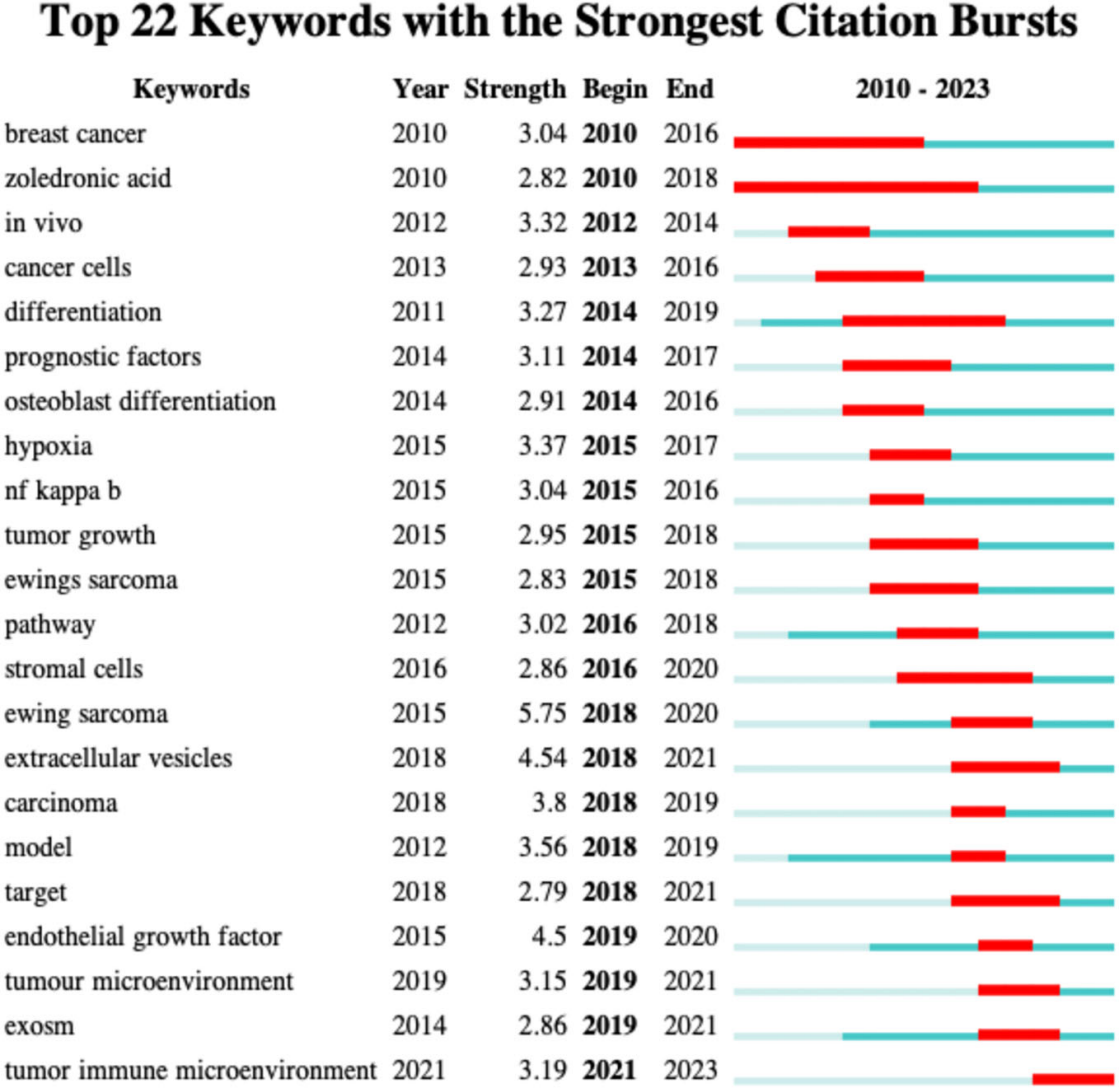
Analysis of Keywords for Explosive Growth in Osteosarcoma and Microenvironment Research, 2010-2023.

From **Figure 10**, we can easily find that the most recent research explosion in this field is the term “tumor immune microenvironment” that will appear in 2021, which also indicates the most likely hot direction and trend in the research field of osteosarcoma and microenvironment now and in the future. We also found that there are a few words in the field that have appeared in recent years or are just starting to fade, such as “extracellular vesicles” which exploded in 2018 and is cooling off in 2021, and “exosm “exosm” is exploding in 2019 and cooling off in 2021. These words have important meanings, and the changes of these words can indicate the previous research direction and current content as well as the possible future hotspots and cutting-edge directions in the field of osteosarcoma microenvironment.

### Most cited literature in published articles in osteosarcoma and microenvironment research, 2010-2023

**Figure 11:**
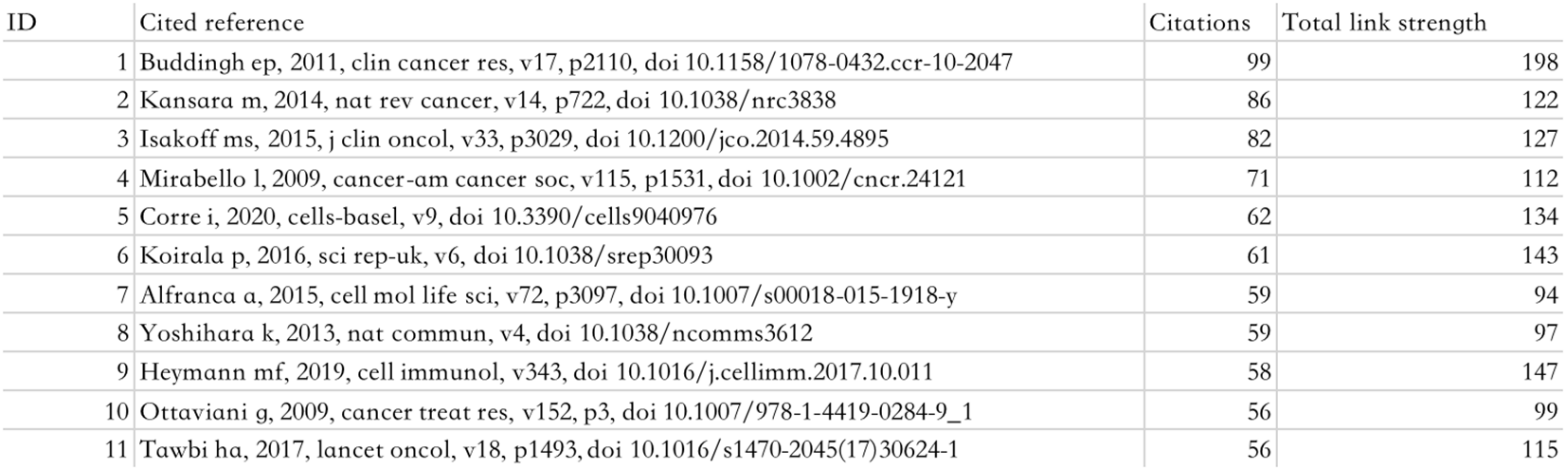
Top 11 Most Cited Literature in Published Articles in Osteosarcoma and Microenvironment Research, 2010-2023.

We found that the most cited paper here examines the relationship between infiltrating macrophages in the tumor microenvironment and osteosarcoma (22), and the quality of the article is very high, so it’s no surprise that a lot of researchers would cite this article. Additional highly cited articles in the field have examined the relationship between immune signaling pathways in osteosarcoma and clinical treatments and molecular mechanisms of osteosarcoma, among others (23, 24). This can be seen in **Figure 11**.

## Discussion

In recent years, we have found that the term “microenvironment” has appeared with increasing frequency in osteosarcoma research, and more studies and basic experiments on the microenvironment of osteosarcoma are underway. Among the many studies in this field, some researchers have investigated the effect of Mesenchymal stem cells (MSCs) in the microenvironment of tumor cells on the development of osteosarcoma (10, 25–27). There have also been studies on the effect of RNA level in the microenvironment of tumor cells on osteosarcoma and the analysis of the mechanism (28, 29). In addition, it has been found that complement C1q may be an index of microenvironmental remodeling in osteosarcoma (30). There are also researchers who suggest that CD8+ T cells can enhance penetration into the microenvironment of osteosarcoma cells (31). It is also enough to show that the direction of scientists’ research aimed at osteosarcoma and the microenvironment is multifaceted and systematic.

We have conducted a great deal of very in-depth analysis to profile and visualize the authors, country regions, and journals of the articles published between January 1, 2010, and August 01, 2023, in the research field related to Osteosarcoma Microenvironment in order to provide in-depth analysis and guidance on potential future hotspots and cutting-edge directions in this field of Osteosarcoma and Microenvironment. We located 821 papers that suit the field’s content using Citespace, VOSviewer, and other software, and we were able to display the research done in this area during the last 13 years. Seven of the top 10 institutions were Chinese universities, while Shanghai Jiao Tong University received the most citations. China is the nation that has been referenced the most in the subject, and England has received the most citations on average.

In terms of authorship, we found that research in the field is roughly divided into 5 core areas, or 5 clusters shown in **Figure 4A**, where Baldini, Heymann, Avnet, and Redini are all important researchers in a more central position. In terms of the countries or regions that have studied the field of osteosarcoma and the microenvironment, our analysis shows that With a combined total of 586 publications published in this subject in these two nations, which accounts for more than 70% of the global research in this field, China and the United States are the two leading research hubs in the field of osteosarcoma metabolism.

Additionally, “microenvironment,” “expression,” “cancer,” “osteosarcoma,” and “metabolism” are all commonly recurring terms in the findings. Additionally, between 2010 and 2023, these phrases essentially denote the most well-liked research trajectories in the area of osteosarcoma and microenvironment. In addition, using a methodology exclusive to Citespace software, we looked at key terms that have had astronomical growth in the industry over the previous 13 years. This word generally refers to how drastically a field of study has changed over a certain amount of time and can help researchers identify hotspots and emerging trends.

In the case of osteosarcoma and the microenvironment, the results show that the key terms that have exploded in recent years are “carcinoma”, “tumor immune microenvironment”, “exosome”, “microenvironment”, “microenvironment”, “exosome”, “exosome”, and “microenvironment”, “model”, and “tumor microenvironment”, with most of these words exploding in frequency from 2017-2023. Additionally, it is possible to forecast that in the upcoming years, these hotspots will likely dominate the field of osteosarcoma and microenvironment study.

We have intrinsic constraints related to particular research pair, but our work is the first bibliometric analysis and visualization in the field of osteosarcoma and the microenvironment. Since using numerous databases simultaneously in this sort of study is challenging, for instance, we only utilized the WoS database, but since the WoS database is the most commonly used, acknowledged, and covered database in bibliometrics (32–35), the results we ultimately produced are relatively convincing, and the results of our study reflect the overall research in the field very well. trends in the field.

## Conclusion

We have analyzed and visualized the bibliometric study of research in the field of osteosarcoma and microenvironment research from 2010 to 2023 using VOSviewer and Citespace and R language software. This work is the first to use bibliometric approaches to comprehensively assess and illustrate the research on osteosarcoma and the microenvironment. We have a very intuitive description and visualization of authors, countries or regions, academic institutions, keywords, and outbreak words in the field of osteosarcoma microenvironment. The field of osteosarcoma microenvironment research is on fire by leaps and bounds, and we hope our research can help. This analysis and visualization will be useful for directing future research in this area.

## Data availability statement

The original contributions presented in the study are included in the article/supplementary material. Further inquiries can be directed to the corresponding author.

## Ethics statement

Ethical approval was not required for the study involving humans in accordance with the local legislation and institutional requirements. Written informed consent to participate in this study was not required from the participants or the participants’ legal guardians/next of kin in accordance with the national legislation and the institutional requirements.

## Author contributions

ZS: Conceptualization, Data curation, Formal Analysis, Funding acquisition, Investigation, Methodology, Project administration, Resources, Software, Supervision, Validation, Visualization, Writing – original draft, Writing – review & editing. WZ: Data curation, Formal Analysis, Methodology, Project administration, Resources, Supervision, Validation, Visualization, Writing – review & editing.
